# Comprehensive fatty acid fractionation profilling in preeclampsia: a case control study with multivariable analysis

**DOI:** 10.1186/s12884-021-04313-3

**Published:** 2022-01-03

**Authors:** Herlambang Herlambang, Anggelia Puspasari, Citra Maharani, Rina Nofri Enis, Susan Tarawifa, Amelia Dwi Fitri, Huntari Harahap, Asro Hayani Harahap, Erny Kusdiyah, Mas Rizky Anggun Adipurna Syamsunarno

**Affiliations:** 1 Departement of Obstetrics and Gynecology, Faculty of Medicine and Health Sciences, Universitas Jambi, Jambi, Indonesia; 2 Departement of Physiology, Faculty of Medicine and Health Sciences, Universitas Jambi, Jambi, Indonesia; 3 Departement of Medical Biology and Biochemistry, Faculty of Medicine and Health Sciences, Universitas Jambi, Jambi, Indonesia; 4 Departement of Anatomy, Faculty of Medicine and Health Sciences, Universitas Jambi, Jambi, Indonesia; 5 Departement of Medical Education, Faculty of Medicine and Health Sciences, Universitas Jambi, Jambi, Indonesia; 6 Departement of Public Health, Faculty of Medicine and Health Sciences, Universitas Jambi, Jambi, Indonesia; 7 Departement of Biomedical Sciences Faculty of Medicine Universitas Padjadjaran, Bandung, Indonesia

**Keywords:** Fatty acid fraction, Omega-3, Omega-6, Palmitoleic acid, Preeclampsia, Saturated fatty acid

## Abstract

**Background:**

Preeclampsia is a complication during pregnancy characterised by new-onset hypertension and proteinuria that develops after 20 weeks of gestation. Dyslipidemia in pregnancy is correlated with an increased risk of preeclampsia. However, the dynamic changes in lipid metabolic product, particularly fatty acid fraction, in preeclampsia maternal circulation, are not well understood. This study aimed to investigate fatty acid fraction in preeclampsia maternal blood compared with normotensive normal pregnancy.

**Methods:**

A total of 34 women who developed preeclampsia and 32 women with normotensive normal pregnancy were included in our case-control study. Maternal blood samples were collected for serum fatty acid fractions analysis and other biochemical parameters. Serum fatty acid fractions included long-chain polyunsaturated fatty acid (LCPUFA), monounsaturated fatty acid (MUFA), saturated fatty acid, and total fatty acid, measured with gas chromatography-mass spectrometry (GC-MS). The mean difference of fatty acid level was analysed using parametric and non-parametric bivariate analysis based on normality distributed data, while the risk of preeclampsia based on fatty acid fraction was analysed using a logistic regression model.

**Results:**

Women with preeclampsia have lower high-density lipoprotein (53.97 ± 12.82 mg/dL vs. 63.71 ± 15.20 mg/dL, *p* = 0.006), higher triglyceride (284.91 ± 97.68 mg/dL vs. 232.84 ± 73.69 mg/dL, *p* = 0.018) than that in the normotensive group. Higher palmitoleic acid was found in women with preeclampsia compared to normotensive normal pregnancy (422.94 ± 195.99 vs. 325.71 ± 111.03 μmol/L, *p =* 0.037). The binary logistic regression model showed that pregnant women who had total omega-3 levels within the reference values had a higher risk of suffering preeclampsia than those with the higher reference value (odds ratio OR (95% CI): 8,5 (1.51–48.07), *p* = 0.015). Pregnant women who have saturated fatty acid within reference values had a lower risk for suffering preeclampsia than those in upper reference value (OR (95% CI): 0.21 (0.52–0.88), *p* = 0.032).

**Conclusion:**

Overall, palmitoleic acid was higher in women with preeclampsia. Further analysis indicated that reference omega-3 in and high saturated fatty acid serum levels are characteristics of women with preeclampsia.

## Introduction

Preeclampsia is a disorder that develops during pregnancy characterised by new-onset hypertension and proteinuria after 20 weeks of gestation and can rapidly progress to severe complications, including maternal and fetal mortality [1, 2]. The mechanisms that initiate preeclampsia have many theories; nevertheless, the disruption of trophoblast invasion during placenta development leads to hypoxia, oxidative stress, inflammation, and further endothelial dysfunction is widely accepted [3, 4]. A recent study reported lipid metabolic alteration involved in preeclampsia pathogenesis. The catabolic state of fatty acid by higher lipolysis due to the rapid growth of fetal development normally occurs during the third semester of pregnancy. This catabolic state is higher in preeclampsia than in normal pregnancy, causing hyperlipidemia. This hyperlipidemia enhances oxidative stress and inflammation and leads to endothelial dysfunction [5–7]. An in vitro study reported an increased lipid droplet accumulation in endothelial cells, conditioned with maternal plasma and replicated after exposure to free fatty acid at the combined concentration, defining preeclampsia. This process enhanced cell apoptosis through a significant decrease of mitochondrial dehydrogenase [8].

Serum fatty acid consists of different fatty acid fractions, including long-chain polyunsaturated fatty acid (LCPUFA), monounsaturated fatty acid (MUFA) and saturated fatty acid (SFA), together with all of their fractions. A lipidomic analysis showed a different role of this fatty acid in the pathogenesis of the disease [9–12]. Omega-3 LCPUFA and omega-6 fraction are precursors of prostanoids and leukotrienes involved in inflammation, vasoconstriction, oxidative stress, and platelet aggregation [11, 13]. Myristic and palmitic acid, members of SFAs, are associated with the risk of hypertension in pregnancy [12]. SFAs may promote pro-coagulation [14] that is associated with placental ischemia [15], and MUFA has a protective role in cardiovascular diseases [16]. However, the information regarding comprehensive fatty acid profiles in pregnancy and preeclampsia is still limited. This study aimed to investigate fatty acid fraction in preeclampsia maternal blood compared with normotensive pregnancy.

## Methods

### Study design

This case-control study aimed to compare the fatty acid fraction in preeclamptic maternal blood to that of normal pregnancy. The protocol was arranged based on the Helsinki Declaration and approved by the Health Research Ethics Committee of the Faculty of Medicine, Public health and Nursing, Universitas Gadjah Mada, Dr. Sardjito Public Hospital, Yogyakarta, Indonesia (Ethical approval number: KE/FK/1189/EC/2020). All the subjects signed informed consent forms after receiving detailed information concerning the purpose of the study. Thirty-four women with preeclampsia and thirty-two women with normotensive pregnancies living in Jambi Province, Indonesia, were recruited. Preeclampsia was defined based on the American College of Obstetricians and Gynecologists (ACOG) 2013 criteria. Inclusion criteria for the preeclampsia group were a pregnant woman who had hypertension (systolic blood pressure ≥ 140 mmHg and or diastolic blood pressure ≥ 100 mmHg) after 20 weeks of pregnancy with one or more of the following conditions: semiquantitative proteinuria > + 1; acute kidney failure sign by creatinine > 1,1 g/dL; serum glutamate oxaloacetate transferase (SGOT); and/or serum glutamate pyruvate transferase (SGPT) > 40 IU/dL; thrombocyte < 100.000 cell/mm^3^. The inclusion criteria for the control group were antepartum normotensive healthy women with a gestational age-matched with the preeclamptic group. The exclusion criteria were twin pregnancy, the mother with signs of active clinical infection, pregnancy with assisted technology for fertilisation, history of kidney and hepatic failure. All the subjects were followed until childbirth. Dyslipidemia defined as as a condition of elevated total cholesterol in blood plasma, low density lipoprotein (LDL), triglycerides, and decreased high density lipoprotein (HDL) according to the national cholesterol education program adult treatment panel (NCEP ATP) III criteria.

Blood pressure data were taken from the highest recorded blood pressure during the first came to the hospital until delivery, regardless of gestational age when the patients first came. The early-onset preeclampsia and late-onset preeclampsia was not differentiated in this study. Protein levels were taken from urine samples and measured with quantitative colourimetric methods. Creatinine serum was measured following an enzymatic-colourimetric procedure. The SGOT, SGPT serum level was measured based on the international federation of clinical chemistry (IFCC) with pyridoxal-5-phosphate activation methods. Thrombocyte count was measured with an automated hemato-analyser.

### Fatty acid quantification

Fatty acid fractionation measurement used blood plasma, which was separated by centrifugation. As much as 5 mL of blood from the antecubital vein was taken from all subjects after 8–10 h of fasting. Gas chromatography-mass spectrometry (GC-MS) was used to measure fatty acid fractionation. The GC-MS was performed based on the methods described in a previous study [17]. The numeric data of the fatty acid fractionation group, based on the Mayo clinic cut off point, were set as an upper reference, reference, and below reference values.

This research quantified the comprehensive fatty acid fraction including long-chain polyunsaturated fatty acid and its fractionation including omega-3, alpha-linolenic acid (ALA), eicosapentaenoic acid (EPA), docosahexaenoic acid (DHA), omega-6, linoleic acid (LA), gamma-linolenic acid (GLA), dihomo-gamma-linolenic acid (DGLA), and arachidonic acid (AA). We also measured the oleic acid and palmitoleic acid as the fraction of monounsaturated fatty acid. Myristic acid, palmitic acid, and stearic acid were also measured as fractionation of saturated fatty acids.

### Data analysis

Numeric data was tested using the Kolmogorov-Smirnov distribution, log 10 used for data transformed. Data that were not normally distributed after being transformed were presented as median (min-max) and evaluated using the Mann-Whitney test. Normally distributed data were tested by independent *t*-test and presented as mean ± SD. Categorical data were analysed bivariate using chi-square or Fisher exact test based on the percentage of expected value. Multivariate analysis with a stepwise binary logistic regression model was used to predict preeclampsia based on the fatty acid fractionation profile. The data was analysed using SPSS version 26.

## Results

Sixty-six pregnant women participated in this study. Baseline subject characteristics and the neonatal outcome is shown in Table 1.

**Table 1:** Baseline subject characteristics

Subject characteristic	Preeclampsia (***n*** = 34)	Normotensive (***n*** = 32)	***P*** value
**Maternal characteristic**			
**Age, years**	29.73 ± 7.08	30.09 ± 7.06	0.838^c^
**Blood Pressure**			
SBP, mmHg	170.0 (140.0–200.0)	110.0 (100.0–130.0)	< 0.001^d*^
DBP, mmHg	100.0 (80.0–160.0)	70.0 (60.0–80.0)	< 0.001^d*^
**Gravidarum, n**			
Primigravida	13	13	0.843^a^
Multigravida	21	19	
**Parity, n**			
Nulliparous	11	13	0.485^a^
Multiparous	23	19	
**BMI**	25.76 (21.93–35.93)	25.86 (18.82–33.31)	0.653^d^
Total cholesterol, mg/dL	243.47 ± 44.32	250.91 ± 47.76	0.514
LDL-cholesterol, mg/dL	139.97 ± 33.48	143.09 ± 42.37	0.740
**HDL-cholesterol, mg/dL**	**53.97 ± 12.82**	**63.71 ± 15.20**	0.006*
**Triglyceride, mg/dL**	**284.91 ± 97.68**	**232.84 ± 73.69**	0.018*
**Neonatal outcome**			
Gestational age, weeks	37.5 (31.0–41.0)	38.0 (28.0–41.0)	0.155^d^
Baby birth weight, gram	2600 (900–4500)	3045 (1400–4690)	0.051^d^
**Baby birth for gestational age, n**			
Small for gestation age	23	27	0.113^a^
Proper for gestational age	11	5	
**APGAR score, n**			
Asphyxia, APGAR < 7	9	1	0.009^b*^
Normal, APGAR ≥7	25	31	

^a^Chi-square test, ^b^Fisher exact test, ^c^*t*-test, ^d^non-parametric test, *p < 0.005: statistically significant. Normally distributed data presented as mean ± standard deviation, transformed not normally distributed data presented as median (min-max). *SBP* Systolic blood preasure, *DBP* Diastolic blood preasure, *BMI* Body mass index, *LDL* Low density lipoprotein, *HDL* High density lipoprotein, *APGAR* Appearance, pulse, grimace, activity, respiration

Normotensive women are older, have higher BMI, and the percentage of primipara and nulliparous is higher than that of the preeclampsia group, but the difference is not statistically significant. The preeclamptic group have a higher systolic and diastolic blood pressure than normotensive women do and are statistically significant (Table 1).

The total cholesterol and LDL cholesterol were slightly different between the preeclampsia and normotensive group. Lower HDL and higher triglyceride are found in women with preeclampsia, and the difference is statistically significant (Table 1).

The control group have longer gestational time, a lower percentage of small babies for gestational age than those of the preeclampsia group, but the difference is not statistically significant. The preeclamptic group have a significantly higher percentage of baby birth with asphyxia than the percentage of the control group (Table 1).

The bivariate analysis of fatty acid profiling is shown in Table 2. The group with preeclampsia has lower HDL and higher triglyceride than the normotensive group do, and both are statistically significant. The omega-6/omega-3 ratio and AA/DHA ratio was higher in the preeclamptic than that of the normotensive group, but this difference is not statistically significant.

**Table 2:** Bivariate analysis of the fatty acid profiling

Fatty acid fractionation	Preeclampsia (***n*** = 34)	Normotensive (***n*** = 32)	***P***-value
LCPUFA,	5750.67 ± 1476.53	5706.87 ± 977.46	0.887
w6/w3 ratio	11.36 ± 3.40	10.94 ± 3.12	0.633
AA/EPA ratio	36.50 (9.00–134.00)	44.00 (10.00–116.0)	0.130^a^
AA/DHA ratio	1.66 ± 0.387	1.49 ± 0.369	0.077
Total Omega-3	494.64 ± 171.00	510.53 ± 157.47	0.618
w3 index	3.00 (2.00–6.00)	4.00 (2.00–5.00)	0.159^a^
ALA (C18:3 w3)	40.79 ± 17.70	40.15 ± 15.61	0.877
EPA (C20:5 w3)	24.67 ± 23.63	18.34 ± 15.88	0.111
DHA (C22:6 w3)	429.26 ± 145.59	451.87 ± 137.81	0.466
Total Omega-6	5240.88 ± 1367.31	5196.37 ± 889.10	0.877
LA (C18:2 w6)	4244.0 ± 1198.49	4278.84 ± 754.12	0.877
GLA (C18:3 w6)	12.23 ± 7.05	10.50 ± 4.80	0.310
DGLA (C20:3 w6)	281.97 ± 90.0	249.93 ± 69.32	0.112
AA (C20:4 w6)	700.02 ± 250.94	657.03 ± 194.62	0.442
Omega-9 (Oleic acid, C18:1 w9)	3075.58 ± 950.43	2784.65 ± 795.29	0.184
Saturated fatty acid	5746.79 ± 1578.68	5442.43 ± 1140.38	0.377
Myristic acid, C14:0	151.08 ± 115.13	166.46 ± 76.30	0.188
Palmitic acid, C16:0	4834.32 ± 1341.59	4504.96 ± 1003.46	0.261
Stearic acid, C18:0	756.17 ± 185.33	774.21 ± 126.48	0.648
Monounsaturated fatty acid	3511.52 ± 1075.18	3102.25 ± 881.53	0.095
**Palmitoleic acid (C16:1 w7)**	**422.94 ± 195.99**	**325.71 ± 111.03**	**0.037**
Oleic acid (C18:1 w9)	3075.58 ± 950.43	2784.65 ± 795.29	0.184
Total fatty acid	14,974.67 ± 4052.2	14,275.59 ± 2883.22	0.420

^a^nonparametric test; otherwise *t*-test, *p < 0.005: statistically significant. Fatty acid fractionation in μmol/L. *AA* Arachidonic acid, *ALA* Alpha-linolenic acid, *DGLA* Dihomo-gamma-linolenic acid, *DHA* Docosahexaenoic acid, *EPA* Eicosapentaenoic acid, *GLA* Gamma-linolenic acid, *LA* Linoleic acid, *LCPUFA* long-chain polyunsaturated fatty acid; w3; omega-3; w6: omega-6

The total LCPUFA, omega-3, and omega-6 are not significantly different between the preeclamptic and normotensive group. Omega-3 fractionation ALA, EPA, DHA and Omega-6 fractionation LA, GLA, DGLA, and AA are also not significantly different between both groups; however, the DHA is higher in the normotensive group while AA is higher in the preeclamptic group (Table 2).

Saturated fatty acid and monounsaturated fatty acid are higher in the group with preeclampsia, but this difference with that of the normotensive group is not statistically significant. Palmitoleic acid as monosaturated fatty acid fractionation is significantly higher in the preeclampsia group compared to that of normotensive women (Table 2).

A stepwise logistic regression model was created, from which the model with the most significant *p*-value was taken. The results show that subjects with total omega-3 blood levels within the reference value have an 8.5x higher risk of suffering preeclampsia than subjects within the upper reference value. Women who have saturated fatty acid within the reference value have as much as 21% lower risk of suffering preeclampsia than the risk of subjects within the upper reference value (Table 3).

**Table 3:** Logistic regression model for fatty acid profiling used to predict preeclampsia

Fatty acid profiling	B	SE	Adjusted ***p***-value	OR (95% CI)
**Total Omega-3**				
Upper reference value (> 500 umol/L)	**ref**			
Reference value (200–500 umol/L)	2.141	0.884	0.015*	8.50 (1.51–48.07)
Below reference value	NS			
**Saturated fatty acid**				
Upper reference value (> 5500 umol/L)	**ref**			
Reference value (2500–5500 umol/L)	−1.540	0.719	0.032*	0.21 (0.52–0.88)
**Stearic acid**	NS			
**EPA**	NS			

The model is a stepwise logistic regression model with the most significant *p*-value. The Hosmer and Lemeshow test was performed to analyse the goodness of fit. The *p* value for all models was > 0.05. B is the logistic regression model coefficient; SE the standard error; OR the odds ratio with 95% confidence interval (CI); ref. was reference genotype; *p* < 0.005: statistically significant

Furthermore, we also used a stepwise regression model to predict small-for-gestational-age baby weight in the group with preeclampsia. The model with the most significant *p*-value was taken (Table 4).

**Table 4:** Logistic regression model for fatty acid profiling to predict small-for-gestational-age baby weight in the preeclampsia group

Fatty acid profiling	B	SE	Adjusted ***p***-value	OR (95% CI)
**Total Omega-3**				
Upper reference value (> 500 umol/L)	**ref**			
Reference value (200–500 umol/L)	2.692	1.305	0.039*	14.76 (1.14–190.37)
**Omega-6/3 ratio**				
Upper reference value (> 10.7)	**ref**			
Reference value (3.4–10.7)	2.718	1.192	0.023*	15.14 (1.46–156.51)
**AA**	NS			

The model was a stepwise logistic regression model with the most significant *p*-value.. The Hosmer and Lemeshow test was performed to analyse the goodness of fit. The *p* value for all models was > 0.05. B is the logistic regression model coefficient; SE the standard error; OR the odds ratio with 95% confidence interval (CI); ref. was reference genotype; *p* < 0.005: statistically significant

The women who have total omega-3 blood levels within the reference value have a higher risk of having a small-for-gestational-age baby weight than those with total omega-3 within the upper reference value. A similar trend is also observed for the omega-6/3 ratio (Table 4).

## Discussion

The baseline subject characteristics of this study were not statistically different based on age, gravid status, and parity status between the preeclamptic and normotensive groups. Higher blood pressure was standard in the group with preeclampsia.

Some differences in BMI, total cholesterol, and LDL cholesterol were found between both groups. Higher plasma triglycerides and lower plasma HDL were found in women with preeclampsia. Pregnancy altered the maternal lipid metabolism; catabolic state signed by increased lipolysis occurred during the third pregnancy. Higher plasma FFA, glycerol, and triglycerides were found as lipolysis by-products and compensation for the higher energy demand due to rapid fetal development. In addition to the increased triglycerides, the triglyceride-rich HDL2 also increased [6, 18]. Lipid metabolic maladaptation in preeclampsia correlated with higher serum triglycerides, increased circulating FFA, and lower HDL than normal pregnancy, which mimics insulin resistant syndrome [19, 20].

A recent study reported the role of FFA in inflammation, oxidative stress, and cell apoptosis pathway, which is involved in the pathogenesis of preeclampsia [10]. This study quantified various fatty acid fractions such as LCPUFA, MUFA, saturated fatty acid, and total fatty acid. The fatty acid fraction may have different impacts on the pathogenesis of preeclampsia.

The fraction of essential omega-3 fatty acid ALA is a precursor of EPA and DHA. Both EPA and DHA can be converted to anti-inflammatory prostanoids and leukotrienes series. Linoleic acid is an essential omega-6 fatty acid, which can be converted to DGLA and AA. The AA is a precursor of pro-inflammatory prostanoids and leukotrienes, and DGLA has anti-inflammatory properties. The prostanoids and leukotrienes derivate from omega-3 and omega-6 are also involved in platelet aggregation, vasoconstriction, and prothrombotic state. All of that contribute to the pathophysiology of preeclampsia. Previous studies have proposed that lower omega-6 serum and higher omega-3 serum levels can decrease the risk of suffering preeclampsia [8, 11, 21]. In accordance with previous studies, this research also reported that subjects who had plasma omega-3 within the reference value had a higher risk of suffering preeclampsia and small-for-gestational age baby weight than those who had plasma omega-3 within the upper reference value. Plasma level of omega-6/3 ratio in the reference value has a higher risk of having a small-for-gestational age baby in the preeclamptic group than those within our study’s upper reference value. Because this result is contrary to previous studies, the exact mechanism of this disorder is not yet clear. Hence further dietary assessments are needed.

Our study reported that the plasma level of saturated fatty acid within reference value was a protective factor for suffering preeclampsia; the mechanism of saturated fatty acid within the upper reference values in preeclampsia remains unclear. An in vitro study using culture cells reported a decreased mitochondrial dehydrogenase activity and increased apoptosis of human umbilical vein after saturated fatty acid exposure [22]. An epidemiology study also found a positive correlation between saturated fatty acids and pregnancy-induced hypertension in preeclampsia [23]. Furthermore, saturated fatty acids may promote pro-coagulation activity [14], associated with placental ischemia [15].

Our study’s main and unique finding was the higher level of palmitoleic acid in preeclamptic group than that of the normotensive group. To the best of our knowledge, no study has investigated the correlation and mechanism of palmitoleic acid in preeclampsia. Palmitoleic acid is a monounsaturated and omega-7 fatty acid derived from the desaturation of palmitic or vaccenic acid, used as a *de-novo* lipogenesis marker. Palmitoleic acid is proposed to be the new lipokine, which influences interorgan metabolic correlation [24]. Previous studies reported higher plasma palmitoleic acid correlated with multiple cardiometabolic risks, including high blood pressure, dyslipidemia, and endothelial dysfunction. Contrary to those studies, lipokine has been reported to decrease insulin resistance and inflammation status [25]. Further research is thus needed to investigate how higher palmitoleic acid in preeclampsia mimicks adipokine resistance in many cardiovascular diseases considering that evidence shows that preeclampsia mimicks insulin resistance syndrome.

In addition, a decreased mitochondria capacity for long-chain beta-oxidation through decreased expression of long-chain 3-hydroxy acyl-CoA dehydrogenase (LCHAD) was found in a preeclampsia animal model. This metabolic shift enhanced oxidative stress [12]. Although not statistically significant, higher total free fatty acid was found in the preeclamptic group compared to that of the normotensive group.

The sample size was not too large, but it was in accordance with the sample size calculation rule. The main finding of this study enriched the knowledge on the role of fatty acids in preeclampsia. In addition, it promises clinical settings as risk screening and arrangement of nutrition patterns for preeclampsia prevention and management. However, further research with a larger sample size, multi-centre, longitudinal design was needed to reveal a more comprehensive finding.

## Conclusion

Palmitoleic acid was higher in women with preeclampsia compared to that of normotensive pregnant women. Further analysis indicated omega-3 serum level as a protective factor in women with preeclampsia, while saturated fatty acid serum level as a risk factor.

## Data Availability

The data sets analysed during the current study are not publicly available due to confidential agreement subjects but are available from the corresponding author on reasonable request.

## Abbreviations

AA: Arachidonic acid
ALA: Alpha-linolenic acid
DGLA: Dihomo-gamma-linolenic acid
DHA: Docosahexaenoic acid
EPA: Eicosapentaenoic acid
GLA: Gamma-linolenic acid
HDL: High density lipoprotein
LA: Linoleic acid
LCPUFA: Long-chain polyunsaturated fatty acid
LDL: Low density lipoprotein
NCEP ATP: National cholesterol education program adult treatment panel
w3: Omega-3
w6: Omega-6
